# A protocol for high‐throughput, untargeted forest community metabolomics using mass spectrometry molecular networks

**DOI:** 10.1002/aps3.1033

**Published:** 2018-04-02

**Authors:** Brian E. Sedio, Cristopher A. Boya P., Juan Camilo Rojas Echeverri

**Affiliations:** ^1^ Smithsonian Tropical Research Institute Apartado 0843‐03092 Balboa, Ancón Republic of Panama; ^2^ Center for Biodiversity and Drug Discovery Instituto de Investigaciones Científicas y Servicios de Alta Tecnología Apartado 0843‐01103 Ciudad del Saber Republic of Panama; ^3^ Department of Biotechnology Acharya Nagarjuna University Nagarjuna Nagar, 522 510 Guntur India

**Keywords:** chemical ecology, liquid chromatography, molecular networking, tandem mass spectrometry, tropical forest ecology, untargeted metabolomics

## Abstract

**Premise of the Study:**

We describe a field collection, sample processing, and ultra‐high‐performance liquid chromatography–tandem mass spectrometry (UHPLC‐MS/MS) instrumental and bioinformatics method developed for untargeted metabolomics of plant tissue and suitable for molecular networking applications.

**Methods and Results:**

A total of 613 leaf samples from 204 tree species was collected in the field and analyzed using UHPLC‐MS/MS. Matching of molecular fragmentation spectra generated over 125,000 consensus spectra representing unique molecular structures, 26,410 of which were linked to at least one structurally similar compound.

**Conclusions:**

Our workflow is able to generate molecular networks of hundreds of thousands of compounds representing broad classes of plant secondary chemistry and a wide range of molecular masses, from 100 to 2500 daltons, making possible large‐scale comparative metabolomics, as well as studies of chemical community ecology and macroevolution in plants.

Innovations in phylogenetics and phylogenomics are rapidly advancing our understanding of the tree of life, enabling the study of macroevolution at unprecedented scales. Despite these developments, the overwhelming diversity of plant secondary metabolites of unknown structure and the taxonomic rarity of any given compound have until recently remained obstacles to comparative metabolomics, the comparison of small‐molecule metabolite profiles, at the large taxonomic scales necessary for the study of macroevolution and community ecology. However, recent advances in tandem mass spectrometry (MS/MS) bioinformatics enable the high‐throughput comparison of the structures of unknown compounds (Wang et al., 2016), making possible comparative metabolomics at scales necessary for the study of chemical community ecology and macroevolution (Sedio, 2017).

The structural comparison of unknown molecules using MS/MS is possible because molecules with similar structures fragment into many of the same substructures. MS/MS spectra can be collected from complex mixtures directly, or with the added separation provided by ultra‐high‐performance liquid chromatograph (UHPLC), making MS‐based metabolomics scalable to data sets containing hundreds of samples and tens of thousands of unique molecules. Comparative metabolomics of plant tissues, individuals, or species is aided by the organization of pairwise MS/MS similarities into molecular networks in which nodes represent compounds and links indicate structural similarity (Watrous et al., 2012; Wang et al., 2016). A comparison of MS/MS spectra of unknown compounds to public spectral libraries, such as with the Global Natural Products Social (GNPS) Molecular Networking platform (https://gnps.ucsd.edu/; Wang et al., 2016), can identify known structures; comparing unknown spectra to each other can facilitate the study of chemical community ecology and evolution, even in diverse and understudied systems like tropical forests (Sedio, 2017; Sedio et al., 2017). The strength of MS/MS molecular networking metabolomics lies in its generality and scalability. Hence, there is a need for a simple, general, and repeatable method for the collection of MS/MS spectra that is broadly inclusive of chemical classes and molecular weights that can unleash the potential of molecular networking bioinformatics for the study of chemical community ecology and evolution.

From 2014 to 2017, we investigated intra‐ and interspecific variation in foliar metabolomes in tree communities in temperate deciduous forest at the Smithsonian Environmental Research Center (SERC) near Edgewater, Maryland, and in tropical moist forest at Barro Colorado Island (BCI), Panama (Sedio et al., 2017; Appendix 1). To facilitate a community‐level comparison of the metabolomic diversity of permanent forest recensus plots at BCI and SERC (B. E. Sedio, J. D. Parker, S. M. McMahon, and S. J. Wright, unpublished data), we generated MS/MS metabolomic data for 613 leaf samples from 204 plant species, resulting in 248,570 individual MS/MS spectra and a molecular network comprising 138,470 consensus spectra, or putative unique molecular structures (Fig. 1; Watrous et al., 2012). An excerpt of this network is presented in Fig. 2 that illustrates the utility of the molecular network approach for (a) visualization of structural relationships among unknown metabolites, (b) comparative metabolomics among plant species, and (c) identification of known compounds by searching public MS libraries (Wang et al., 2016). Here, we describe a protocol for sample collection, chemical extraction, UHPLC‐MS/MS instrumental methods, and bioinformatics workflow for the generation of molecular networks for plant metabolomics. This protocol is simple to execute, broadly inclusive of plant secondary chemical variation, effective over a relatively wide range of variation in polarity and molecular mass, and scalable to sample sizes large enough to facilitate chemical community ecology in species‐rich plant communities such as tropical forests.

**Figure 1:**
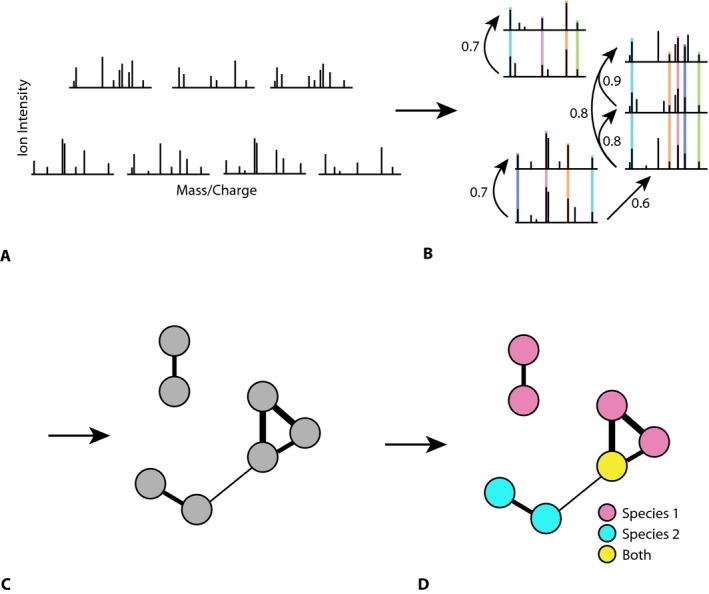
The generation of molecular networks based on mass spectrometry. (A) Tandem mass spectrometry provides fragment ion (MS ^2^) spectra representing seven compounds, with each peak representing the mass‐to‐charge ratio (*m/z*, horizontal axis) and ion intensity (vertical axis) of a constituent molecular fragment. (B) Spectra are aligned (colored vertical lines identify shared molecular fragments), and similarity scores (numbers with arrows) are calculated between every pair. (C) The similarity scores are used to define molecular networks in which nodes represent compounds and the width of the links represents structural similarity. (D) Compounds are mapped onto two plant species. The figure is adapted from Watrous et al. (2012) with permission.

**Figure 2:**
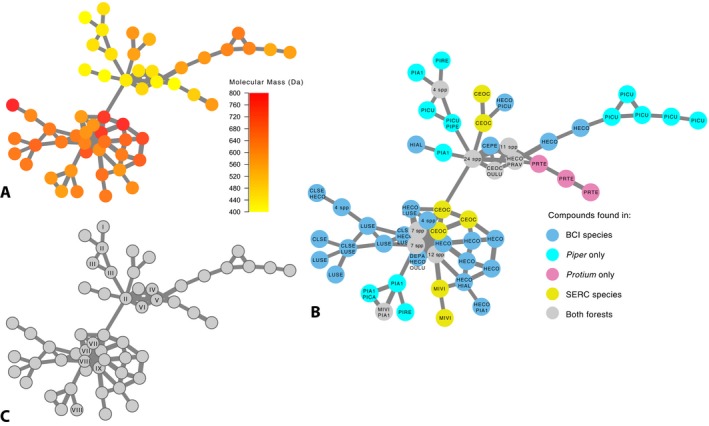
Subset of a molecular network of foliar metabolomes of 204 plant species from Maryland and Panama. Presented is a cluster of 56 nodes that is part of a larger network data set comprising 138,470 consensus spectra, or putative unique molecular structures, derived from 204 plant species (B. E. Sedio, J. D. Parker, S. M. McMahon, and S. J. Wright, unpublished data). (A) The molecular mass of parent ions prior to fragmentation is indicated by a color scale from yellow (400 Da) to red (800 Da). (B) Compounds (nodes) found exclusively in plant species collected at Barro Colorado Island (BCI), Panama, are indicated in blue, and those exclusively found in the tropical tree genera *Piper* and *Protium* are indicated in light blue and pink, respectively. Compounds found exclusively in plant species collected at the Smithsonian Environmental Research Center (SERC) in Maryland, USA, are indicated in yellow, and compounds found in species from both forest sites are indicated in gray. Species codes are CEOC (*Celtis occidentalis*, Cannabaceae, SERC), CEPE (*Ceiba pentandra*, Malvaceae, BCI), CLOC (*Clidemia octona*, Melastomataceae, BCI), CLSE (*Clidemia septuplinervia*, Melastomataceae, BCI), DEPA (*Desmopsis panamensis*, Annonaceae, BCI), HECO (*Heisteria concinna*, Olacaceae, BCI), HIAL (*Hieronyma alchorneoides*, Euphorbiaceae, BCI), LUSE (*Luehea seemannii*, Malvaceae, BCI), MIVI (*Microstegium vimineum*, Poaceae, SERC), OULU (*Ouratea lucens*, Ochnaceae, BCI), PIA1 (*Piper arboreum*, Piperaceae, BCI), PICA (*Piper schiedeanum*, Piperaceae, BCI), PICU (*Piper colonense*, Piperaceae, BCI), PIIM (*Piper imperialis*, Piperaceae, BCI), PIPE (*Piper perlasense*, Piperaceae, BCI), PIRE (*Piper reticulatum*, Piperaceae, BCI), PRAV (*Prunus avium*, Rosaceae, SERC), and PRTE (*Protium tenuifolium*, Burseraceae, BCI; see Appendix 1). (C) Spectra that matched an annotated spectrum in a public library are indicated. Compounds matched in Global Natural Products Social (GNPS) public libraries are: (I) orientin, (II) vitexin, (III) ReSpect:PM007805 isoorientin, (IV) ReSpect:PS086308 orientin, (V) ReSpect:PS043007 puerarin, (VI) ReSpect:PM007810 3′‐O‐Methylluteolin 6‐C‐glucoside, (VII) pentoside of (iso)vitexin, (VIII) hexanoside of (iso)vitexin, and (IX) Massbank:PB006223 vitexin‐2″‐O‐rhamnoside.

## METHODS AND RESULTS

### Field collection

For community metabolomics of the forest plots at BCI and SERC, we collected young, unlignified leaves from saplings encountered in the shaded understory during the rainy season between June and August 2014. Leaves were placed on ice immediately in the forest and transferred to a −80°C freezer within 3 h of collection. See field collection protocol in Appendix 2.

### Extraction and sample preparation

We homogenized 100 mg of frozen leaf tissue on liquid nitrogen in a ball mill (TissueLyser; QIAGEN, Hilden, Germany) and extracted the homogenate with 700 μL of 90% methanol : 10% water (pH 5) for 10 min. Methanol is an effective solvent for small molecules representing a wide range in polarity; mild acidity improves the extraction of most alkaloids. The solution was vortexed and centrifuged, and the supernatant was isolated. The extraction was repeated on the remaining sample, and the fractions were combined. Samples were diluted in identical extraction solvent and filtered using 4‐mm syringe filters with a hydrophilic polytetrafluoroethylene (PTFE) membrane with a 0.20‐μm pore size (Merck Millipore, Billerica, Massachusetts, USA) prior to analysis using UHPLC‐MS/MS. See the chemical extraction protocol in Appendix 3.

### Liquid chromatography instrument methods

Samples were analyzed using an Infinity 1290 UHPLC from Agilent Technologies (Santa Clara, California, USA) with a Kinetex C18 column that was 100 mm in length, 2.1 mm in internal diameter, with a 1.7‐μm particle size (Phenomenex, Torrance, California, USA), and a flow rate of 0.5 mL/min at 25°C (no flow splitting was used prior to infusion into the mass spectrometer). To separate a complex mixture with molecules separated by a wide range of polarity, we employed a 37‐min solvent gradient with 0.1% formic acid in water (A) and 0.1% formic acid in acetonitrile (B): 0–2 min at 5% B, 2–27 min gradient from 5% B to 100% B, followed by 8 min at 100% B, and 35–37 min from 100% B to 5% B.

### Mass spectrometry instrument methods

Separation by LC was followed by electrospray ionization (ESI) in positive mode and MS/MS detection on a micrOTOF‐QIII quadrupole‐time‐of‐flight mass spectrometer (Bruker Daltonics, Billerica, Massachusetts, USA). We optimized MS parameters to detect and fragment molecules representing as wide a range in the mass‐to‐charge ratio (*m/z*) of the parent compound as possible. We began the process of optimization by analyzing ESI‐L low concentration tuning mix (G1969‐85000; Agilent Technologies), as well as foliar extracts of species of *Psychotria* L. (Rubiaceae), a genus that exhibits diverse alkaloids, flavonoids, and terpenes (Riba et al., 2003; Kowalczuk et al., 2015; Klein‐Júnior et al., 2016). Of particular importance was *P. acuminata* Benth., one of the most chemically diverse species known on BCI (Sedio et al., 2017). We analyzed tuning mix and *Psychotria* samples with the Bruker default “tune wide” parameter setting and following Garg et al. (2015). We then sequentially tuned ion guide funnels and multipoles by modifying radio frequency (RF) stepping and transfer time until we were able to detect molecules ranging from 100 to 2500 *m/z*. Data‐dependent collision energies were optimized to improve fragmentation quality and sensitivity.

Mass spectra were acquired using a micrOTOF‐QIII mass spectrometer from Bruker Daltonics by ESI in positive mode. The ESI source parameters were: end plate offset, 500 V; capillary voltage, 4500 V; nebulizer, 2.0 bar (nitrogen gas); dry gas, 9.0 L/min; and dry temperature, 200°C. The ion optics settings included: funnel 1 RF amplitude, 150 Vpp; funnel 2 RF amplitude, 300 Vpp; hexapole RF amplitude, 150 Vpp; in‐source collision‐induced dissociation (isCID) energy, 0.0 eV; quadrupole ion (transfer) energy, 10.0 eV; quadrupole low mass cut‐off, 50.0 *m/z*; and pre‐pulse storage, 10.0 μs. Data were acquired both for molecular ions (MS^1^) and fragment ions (MS^2^) in data‐dependent fragmentation (auto MS/MS). For MS^1^ acquisition, three spectra were collected per second (3 Hz).

For MS^2^ acquisition, the rate of acquisition was slowed down for low‐intensity molecular ions (20,000 counts) to 2 Hz in an attempt to increase the sensitivity for these ions and kept at 3 Hz for high‐intensity molecular ions (1,000,000 counts); we employed a linear gradient in the rate of acquisition for species of intermediate ion intensity. In an attempt to increase sensitivity, we utilized the advanced stepping mode to preferentially transfer (through the collision cell) low‐intensity precursor ions and different fragment ions, resulting in acquisition of an averaged mass spectrum with four different parameter combinations (1: collision RF amplitude, 200 Vpp; transfer time, 96 μs; 2: collision RF amplitude, 300 Vpp; transfer time: 96.0 μs; 3: collision RF amplitude, 580 Vpp; transfer time, 120 μs; 4: collision RF amplitude, 680 Vpp; transfer time, 120 μs), each with an equal percentage of the time allotted for each MS^2^ acquisition cycle.

Data‐dependent fragmentation (auto MS/MS) was set to select a maximum of five precursor ions with intensities ≥6500 counts per fragmentation cycle of 3.0 s. A maximum of three spectra were collected for each precursor ion before placing it in an exclusion list for 1 min to allow collection of as many different ions per chromatographic peak as possible. The fragmentation energies used for two possible charged states (singly and doubly charged) are presented in Table 1.

**Table 1:** Isolation‐ and collision‐induced energies used in data‐dependent fragmentation (auto tandem mass spectrometry) experiments

Mass‐to‐charge ratio (*m/z*)	Isolation width (Da)	Collision energy (eV)	Charge state
100.00	4.00	15.0	1
100.00	8.00	11.3	2
300.00	5.00	20.0	1
300.00	10.00	15.0	2
500.00	6.00	25.0	1
500.00	12.00	18.8	2
1000.00	7.00	35.0	1
1000.00	14.00	26.3	2
1500.00	8.00	47.5	1
1500.00	16.00	35.6	2

The optimized MS method provided a detection range of 100 to 2500 *m/z*. It should be noted that these parameter values are unique to the micrOTOF‐QIII instrument. However, we suggest a similar approach to optimization for a wide mass range by using a calibration solution (e.g., ESI‐Tunemix, G1969‐85000; Agilent Technologies) to sequentially modify the MS settings until the desired *m/z* range is achieved. A chemically diverse biological extract consisting of a single sample (e.g., *P. acuminata*) or a pool of samples that are representative of molecular families of interest can be used to further tune the collision energies and confirm their suitability for the biological system to be analyzed. Although we used static collision energies for discrete *m/z* ranges (Table 1), ramping or stepping the collision energy applied during collision‐induced dissociation within each *m/z* range may further improve the quality of molecular fragmentation achieved over a range of masses, molecular ion stabilities, and chemical classes. To eliminate non‐informative fragmentation spectra, we filtered spectral matches by requiring a minimum number of matched fragment ions in the downstream bioinformatics analyses (see Bioinformatics, below).

For calibration, an external calibration with ESI‐Tunemix (G1969‐85000; Agilent Technologies) or a 10 mM sodium formate solution (in 50 : 50 propan‐2‐ol : water with 0.2% formic acid, v/v) was performed every 12 h using the “Quadratic + HPC” calibration mode. At the same time, depending on availability, reserpine (43530‐4.5ML‐F; Sigma Aldrich, St. Louis, Missouri, USA) or hexakis (1H,1H,2H‐difluoroethoxy)phosphazene (8H79‐3‐02; Synquest Laboratories, Alachua, Florida, USA) was used for post‐acquisition internal calibration. Acquired spectra were internally calibrated and exported in batch mode with Compass DataAnalysis 4.1 SR1 from Bruker Daltonics; refer to Appendix 4 for further details.

### Bioinformatics

We generated a molecular network using the online workflow at GNPS (https://gnps.ucsd.edu/; Wang et al., 2016). First, we filtered the data by removing all MS/MS peaks within ±17 Da of the precursor *m/z*. We then window‐filtered the MS/MS spectra by choosing only the top six peaks in each ±50 Da window throughout the spectrum. The data were then clustered with MS‐Cluster (Frank et al., 2008) with a parent mass tolerance of 2.0 Da and an MS/MS fragment ion tolerance of 0.5 Da to generate consensus spectra representing putative unique molecular structures. Consensus spectra containing <2 spectra were discarded and the remaining spectra were networked. Edges were formed for spectral matches with cosine score ≥0.6 and ≥6 matched peaks. Edges were retained in the network only if both nodes linked by the edge were in each other's top 10 most similar nodes.

During network generation, spectra were compared to annotated spectra in public libraries through GNPS (Wang et al., 2016). We applied identical filter criteria to library spectra as to our input data and employed the GNPS analog library search method with a maximum mass shift of 100 Da. We retained matches to library spectra characterized by a cosine score ≥0.6 and with ≥6 matched peaks. The “group mapping” feature of GNPS allows one to track the origin of spectra, and hence, the plant species, tissue, or treatment in which a compound was detected. Network visualization software such as Cytoscape (http://www.cytoscape.org) can be used to generate publication‐quality figures of molecular networks that illustrate attributes of the data such as molecular mass (Fig. 2A); incidence in plant species, tissues, or treatments (Fig. 2B); and matches with annotated spectra from public MS libraries (Fig. 2C; Wang et al., 2016). A GNPS bioinformatics workflow can be found in Appendix 5.

Recent developments in the bioinformatics pipeline for the assembly of molecular networks have improved upon the methods we describe above in several key respects (Olivon et al., 2017). Namely, the MS‐Cluster algorithm (Frank et al., 2008) for grouping spectra into consensus spectra was originally designed for proteomics rather than small molecule metabolomics and therefore was not designed to consider differences in LC retention time that typically distinguish structural isomers with identical molecular masses. Olivon et al. (2017) describe a bioinformatics workflow that integrates the MS analytical software MZmine 2 (Pluskal et al., 2010) into the workflow for the assembly of raw MS/MS spectra into molecular networks with GNPS. In the short term, we recommend preprocessing MS/MS data using MZmine 2 prior to GNPS network assembly (without using MS‐Cluster) as described by Olivon et al. (2017) to resolve isomeric compounds, annotate molecular networks with putative chemical formulas, and improve the quantification of variation in ion abundances among samples. Future versions of GNPS will incorporate MZmine 2 into the online bioinformatics workflow (M. Wang, University of California, San Diego, personal communication).

## CONCLUSIONS

We have developed an effective untargeted plant metabolomics workflow for community metabolomics, including a protocol for tissue collection in the field, a chemically general extraction protocol that retains compounds from a broad spectrum of plant secondary chemistry and is appropriate for diverse taxa, a UHPLC‐MS/MS instrumental method suitable for a wide range of polarities and molecular size classes, and a protocol for sharing and networking MS/MS data with the GNPS molecular networking platform (Wang et al., 2016). Because of its simplicity and generality, this workflow can be scaled for the collection of large and taxonomically and chemically diverse data sets, such as ecological communities or evolutionary lineages, thus facilitating the study of chemical community ecology and macroevolution (Sedio, 2017). Future efforts should test the robustness of this workflow for field collections in remote locations where the freezing of tissue may be unfeasible and in situ drying of tissue may be the preferred means of sample collection. In addition, alternative extraction solvents, LC column stationary phases, and ionization methods should be explored as these may facilitate the analysis of chemical classes for which our protocol is suboptimal.

## Figures and Tables

**Figure A4‐1:**
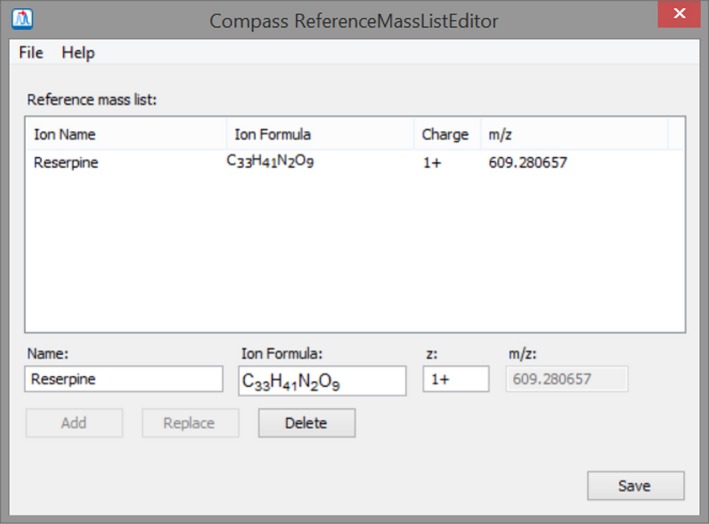
An example lock mass calibrant reference list using reserpine as an internal calibrant.

**Figure A5‐1:**
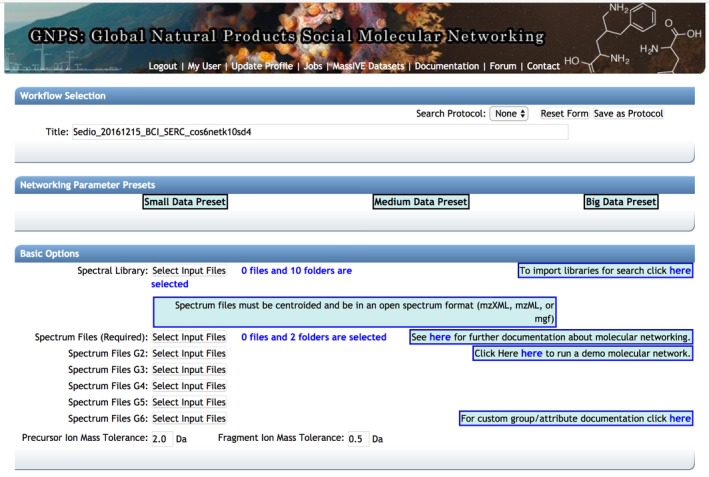
The GNPS *Network Workflow* window.

**Figure A5‐2:**
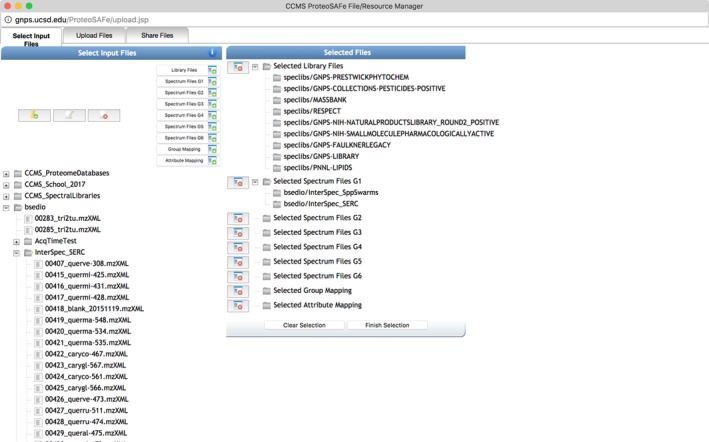
The GNPS *Select Input Files* window.

**Figure A5‐3:**
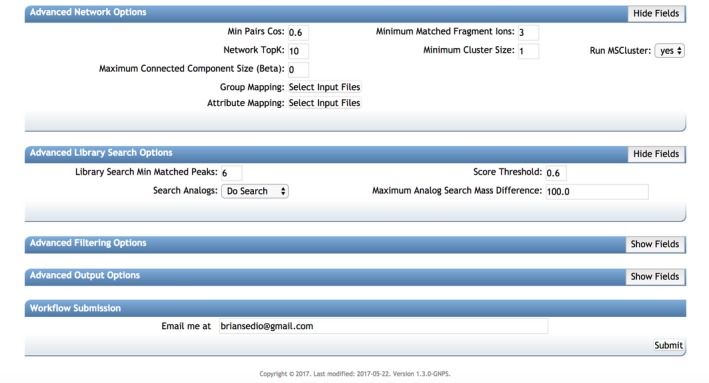
The *Advanced Network Options* and *Advanced Library Search Options* parameter windows on the GNPS *Network Workflow* page.

**Figure A5‐4:**
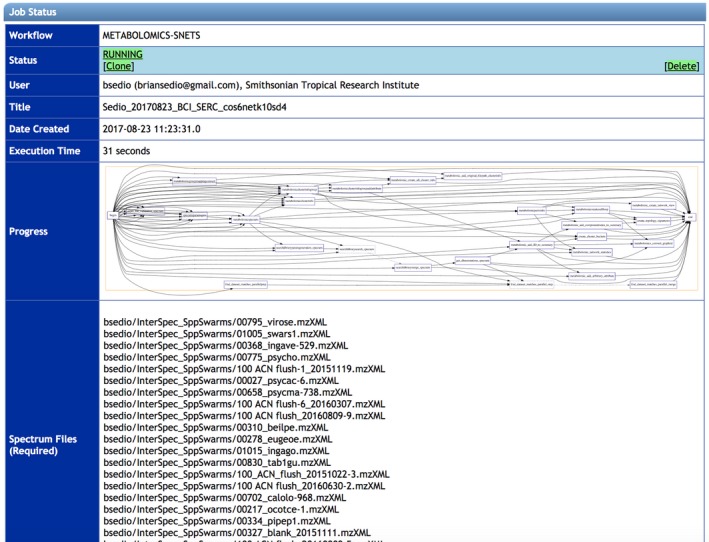
The *Job Status* window during the execution of a network run.

**Figure A5‐5:**
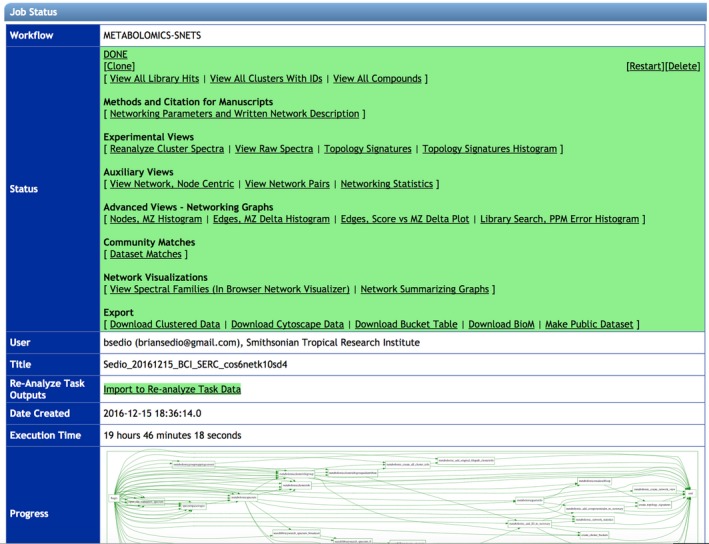
The GNPS *Job Status* window upon completion of a network.

## Appendix 1 Voucher information for species presented in this study.

**Table:**

Species	Family	Voucher specimen accession no.^a^	Collection locality^b^	Herbarium^c^
*Ceiba pentandra* (L.) Gaertn.	Malvaceae	15420	BCI	SCZ
*Celtis occidentalis* L.	Cannabaceae	362	SERC	SERC
*Clidemia octona* (Bonpl.) L. O. Williams	Melastomataceae	15177	BCI	SCZ
*Desmopsis panamensis* (B. L. Rob.) Saff.	Annonaceae	15113	BCI	SCZ
*Heisteria concinna* Standl.	Olacaceae	16017	BCI	SCZ
*Hieronyma alchorneoides* Allemão	Euphorbiaceae	15144	BCI	SCZ
*Luehea seemannii* Triana & Planch.	Malvaceae	15160	BCI	SCZ
*Microstegium vimineum* (Trin.) A. Camus	Poaceae	431	SERC	SERC
*Ouratea lucens* (Kunth) Engl.	Ochnaceae	15136	BCI	SCZ
*Piper arboreum* Aubl.	Piperaceae	15197	BCI	SCZ
*Piper schiedeanum* Steud.	Piperaceae	15167	BCI	SCZ
*Piper colonense* C. DC.	Piperaceae	1172	BCI	SCZ
*Piper reticulatum* L.	Piperaceae	15201	BCI	SCZ
*Prunus avium* (L.) L.	Rosaceae	352	SERC	SERC
*Protium tenuifolium* Engl.	Burseraceae	15262	BCI	SCZ
*Psychotria acuminata* Benth.	Rubiaceae	15334	BCI	SCZ

a Vouchers of these species (not necessarily the individuals sampled in this study) are collected and maintained by the Smithsonian Institution Forest Global Earth Observatory (ForestGEO)–Center for Tropical Forest Science (CTFS). Given are barcode accession numbers.

b Collections were made within ForestGEO‐CTFS forest dynamics plots at Barro Colorado Island (BCI), Panama (9°9ʹN, 79°51ʹW), and at the Smithsonian Environmental Research Center (SERC), Maryland, USA (38°53ʹN, 76°33ʹW).

c Herbarium codes refer to the Summit Herbarium at the Smithsonian Tropical Research Institute (SCZ) and the herbarium at SERC.

## Appendix 2 Two alternative setups for field collection on ice or liquid nitrogen.

- Setup 1: Collection on ice.

This setup is convenient if tissue for metabolomic analysis is intended for interspecific comparative metabolomics, if tissue for metabolomic analysis is to be collected alongside tissue intended for DNA extraction, and if collections are to be at a site within a few hours hike from a laboratory equipped with a −80°C freezer.

A Field supplies:
  1. Plant press, straps, cardboard, blotting paper, and newspaper
  2. Coin envelopes for seeds or small fruits of voucher specimens
  3. GPS unit and maps
  4. Fine‐tip Sharpie marker (Sanford L.P., Downers Grove, Illinois, USA), pencils
  5. Field notebook
  6. Field guide and keys
  7. Hand lens
  8. Hedge clippers
  9. 2‐mL Safe‐Lock tubes (Eppendorf, Hamburg, Germany), two per sample
  10. Rite‐in‐the‐Rain paper (JL Darling, Tacoma, Washington, USA), cut with scissors into rectangles small enough to fit into the 2‐mL tubes
  11. Fabric lunch cooler
  12. Rechargeable ice packs (Thermos, Chicago, Illinois, USA)
  13. Cryogenic gloves
B Field procedure:
  1. Remove plant material sufficient for chemical and DNA extractions and voucher material. Choose material with mature flowers and fruits for voucher specimens. For samples intended for chemical analysis, consider variables such as herbivore damage, leaf ontogenetic stage, individual ontogenetic stage, and light environment when considering which individuals and tissues to sample.
  2. Label two 2‐mL Safe‐Lock tubes for each sample. Place 50 to 100 mg of leaf tissue from a single individual plant into each tube.
  3. Label one small piece of Rite‐in‐the‐Rain paper per tube and place the paper labels in each tube. Add the paper labels to the tubes after the sample plant tissue so that the label can be checked without removing the plant tissue.
  4. Screw on the screw caps and place the tubes into the cooler.
  5. Press three to five voucher specimens for each collection. Record collection date, collector, GPS coordinates, descriptive location, habitat (including light environment and topography), plant habit, reproductive status, color, and other specimen information. See Gostel et al. (2016) for additional information on vouchers.
  6. Upon arrival in the laboratory, label cardboard freezer storage boxes prior to use. Place samples into labeled freezer boxes for storage. Place the freezer boxes into the −80°C freezer using cryogenic gloves.
  7. Check newspaper pressed with voucher specimens daily and exchange for fresh newspaper if saturated with moisture.

II. Setup 2: Collection with liquid nitrogen.

This setup is convenient if tissue for metabolomic analysis is intended for intraspecific or intra‐individual comparative metabolomics, if tissue for metabolomic analysis is to be collected alongside tissue intended for RNA extraction, or if collections are to be made over multiple days at a site more than a few hours from a laboratory equipped with a −80°C freezer.

A Field supplies: In addition to the supplies listed for Setup 1, also bring:
  1. Large liquid nitrogen container, 25 to 50 L (see Yang et al., 2017)
  2. 10‐L cryogenic liquid nitrogen container with straps and carry bag and a holding time of 88 days (SKU YDS‐10; Hardware Factory Store, Los Angeles, California, USA)
  3. Long metal tongs (e.g., VWR 82027‐366; VWR, Radnor, Pennsylvania, USA)
B Field procedure:
  1. Remove plant material sufficient for chemical and DNA extractions and voucher material. Choose material with mature flowers and fruits for voucher specimens. For samples intended for chemical analysis, consider variables such as herbivore damage, leaf ontogenetic stage, individual ontogenetic stage, and light environment when considering which individuals and tissues to sample.
  2. Label two 2‐mL Safe‐Lock tubes for each sample. Place 50 to 100 mg of leaf tissue from a single individual plant into each tube.
  3. Label one small piece of Rite‐in‐the‐Rain paper per tube and place the paper labels in each tube. Add the paper labels to the tubes after the sample plant tissue so that the label can be checked without removing the plant tissue.
  4. Screw on the screw caps and place the tubes into the backpack‐portable liquid nitrogen dry shipper.
  5. Press three to five voucher specimens for each collection. Record collection date, collector, GPS coordinates, descriptive location, habitat (including light environment and topography), plant habit, reproductive status, color, and other specimen information. See Gostel et al. (2016) for additional information on vouchers.
  6. Upon arrival at the camp or vehicle, use cryogenic gloves and long metal tongs to remove sample tubes from the backpack‐portable liquid nitrogen dry shipper and place them into the large liquid nitrogen tank for short‐term storage and transport to the laboratory.
  7. Upon arrival at the laboratory, label cardboard freezer storage boxes prior to use. Place samples into labeled freezer boxes for storage. Place the freezer boxes into the −80°C freezer using cryogenic gloves.
  8. Check newspaper pressed with voucher specimens daily and exchange for fresh newspaper if saturated with moisture.

## Appendix 3 Chemical extraction and sample preparation.

A Tools and equipment
  1. Access to a fume hood during the entire duration of extraction
  2. pH meter. We currently use Mettler Toledo (Columbus, Ohio, USA).
  3. Tissue homogenizer. We currently use QIAGEN TissueLyser (QIAGEN, Hilden, Germany).
  4. Two Styrofoam shipping boxes with lids
  5. Waste beaker
  6. Liquid nitrogen
  7. Benchtop liquid nitrogen container
  8. Centrifuge
  9. Pipettes: 1000 and 200 μL
  10. Pipettes: 10 mL
  11. Graduated cylinders: 500 and 50 mL
  12. Glass bottle: 500 mL
  13. Vortexer
  14. Magnetic stir bar
  15. Stir plate
B Reagents
  1. Ultra‐high‐performance liquid chromatography–tandem mass spectrometry (UHPLC‐MS)‐grade methanol (14262, Honeywell Burdick and Jackson, Muskegon, Michigan, USA)
  2. UHPLC‐MS‐grade water (14263, Honeywell Burdick and Jackson)
  3. Hydrochloric acid solution, 0.05 N (35320, Honeywell Burdick and Jackson)
C Consumables
  1. Kimwipe (Kimberly‐Clark Professional, Roswell, Georgia, USA)
  2. Paper towels
  3. Weigh paper
  4. Pipette tips: 1000 and 200 μL
  5. Stainless steel balls
  6. Microcentrifuge tubes
  7. Disposable nitrile and latex gloves
  8. 4‐mm syringe filters with a hydrophilic polytetrafluoroethylene (PTFE) membrane with 0.20‐μm pore size (Merck Millipore, Billerica, Massachusetts, USA)
D General considerations for working with the TissueLyser and centrifugation (modified from QIAGEN TissueLyser and DNeasy manuals).
  1. Do not allow the metal TissueLyser adaptor plates to come into contact with liquid nitrogen. Expose each plastic tube rack to liquid nitrogen prior to fitting the tube rack into the adaptor plate.
  2. Stainless steel beads are reusable. Wash the beads with warm, soapy water and rinse thoroughly with distilled water to remove soap residue and allow to air dry. If beads are to be used in nucleic acid extractions, incubate beads in 0.4 M HCl for 1 min at room temperature, rinse thoroughly with distilled water, and allow to air dry.
  3. All centrifugation steps should be performed at room temperature.
E Safety
  1. Methanol is highly flammable and toxic. Work in the fume hood and temporarily dispose of tips and tubes in the hood in a resealable bag. Consult the Material Safety Data Sheet (MSDS) for additional information on methanol safety and disposal.
  2. HCl is a strong acid and very hazardous in case of contact with the skin, eyes, or mucous membranes. For these reasons, diluted HCl aqueous solution is preferable to solid HCl or concentrated liquid for reagent preparation in this protocol. Consult the MSDS for additional information on HCl safety and disposal.
F Reagent preparation: Extraction solvent: 500 mL of 90 : 10 methanol : water, pH 5
  1. Pipette 6.7 mL 0.05 N HCl solution into 43.3 mL of UHPLC‐MS‐grade water to create a 0.0067 N HCl, pH 5, stock solution.
  2. In a graduated cylinder, measure 360 mL of UHPLC‐MS‐grade methanol. Add it to a clean 500‐mL bottle.
  3. In a graduated cylinder, measure 40 mL of the 0.0067 N, pH 5, HCl solution. Add it to the 500‐mL bottle containing the methanol.
  4. Stir the solution using a magnetic stir bar on a stir plate prior to use.
G Organic molecule extraction
  1. Work with 12 to 24 samples at a time, including one blank. The blank is a 2‐mL Safe‐Lock tube containing no leaf tissue. Apply all steps to the blank as if it were a leaf sample. Compounds found in blanks will be removed from downstream analyses.
  2. Weigh 100 mg of frozen leaf tissue. Record the weight.
  3. If the sample was collected directly into a 2‐mL Safe‐Lock tube, return the sample to the tube. If sample material was not collected directly into a 2‐mL Safe‐Lock tube, label a tube and place the sample into it.
  4. Place a stainless steel bead into each tube. Screw on the screw cap and place the tube in the TissueLyser tube rack.
  5. Repeat Steps 2 through 4 for a set of 12 or 24 samples.
  6. Cool the TissueLyser tube rack in liquid nitrogen. If using dried or lyophilized tissue, the tubes do not need to be frozen in liquid nitrogen.
  7. Fit each tube rack between the TissueLyser adapter plates and place them into the TissueLyser clamps as described in the TissueLyser User Manual. Tighten the clamps tightly by hand. Work quickly so that the plant material does not thaw.
  8. Grind the samples for 2 min at 20 Hz.
  9. Remove and disassemble the plates and racks, noting the orientation of the tube racks during the first round of homogenization. Ensure that each tube's screw cap is tightly closed.
  10. Cool the tube racks again in liquid nitrogen. Knock the racks upside down against the bench five times to ensure that all stainless steel beads can move freely within the tubes. Ensure that no liquid nitrogen remains, but do not allow the leaf material to thaw.
  11. Grind the samples for another 2 min at 20 Hz.
  12. Remove the plates from the TissueLyser and remove the adapter plates from each tube rack. Knock the racks against the bench five times to ensure that no tissue powder remains in the caps. Keep the samples frozen until extraction solvent is added (Step 13).
  13. Add 700 μL of extraction solvent to each tube.
  14. Vortex each tube for 30 s.
  15. Centrifuge for 3 min at 12,000 rpm.
  16. From each tube, remove 500 μL of supernatant to a fresh, labeled 2‐mL microtube. Be careful not to disturb the layer of solid material at the bottom of the tube.
  17. Add 500 μL of extraction solvent to each tube.
  18. Vortex each tube for 30 s.
  19. Centrifuge for 3 min at 12,000 rpm.
  20. From each tube, remove 500 μL of supernatant to the same, labeled 2‐mL microtube as the first fraction. Be careful not to disturb the layer of solid material at the bottom of the tube.
  21. To prepare a 10× dilution, vortex one tube for 30 s and remove 50 μL to a labeled microtube containing 450 μL of extraction solvent.
  22. To prepare a 100× dilution, vortex the 10× dilution for 30 s and remove 50 μL to a labeled microtube containing 450 μL of extraction solvent.
  23. To prepare the diluted samples for LC‐MS, for each sample, draw the full sample volume into a clean syringe. Remove the syringe needle and replace it with a 4‐mm, 0.20‐μm pore size syringe filter. Express the sample through the filter into a fresh, labeled HPLC vial. Cap the vial.
  24. Discard the syringe filter and replace it with the syringe. To clean the syringe, draw >700 μL methanol into the syringe and express it into a waste receptacle.
  25. Repeat Steps 23 and 24 for all samples to be analyzed by LC‐MS.

## Appendix 4 Calibration and data conversion from .d format to .mzxml format.

*DataAnalysis* 4.1 allows the user to process large batches of data so they can be exported externally. Data processing and export require as input parameters the internal calibrant signal to be used for lock mass calibration and export file type. This protocol requires that *DataAnalysis* and *CompassXport* are installed on the computer used for processing.

A Export data with 32‐bit precision and using recalibrated spectra
  1. Execute *RegEdit* command in the Windows search window.
  2. Locate *HKEY_CURRENT_USER\Software\Bruker Daltonik\CompassXport* folder.
  3. Double‐click *ExportPrecision64Bit*.
  4. Set *Value data* as *0*.
  5. Click *OK*. This will ensure that *.mzXML* files are exported with 32‐bit precision.
  6. In the same registry folder, double‐click *UseRecalibratedSpectra*.
  7. Set *Value data* as *1* (or make sure this was the value set by default).
  8. Click *OK*. This will ensure that the spectra exported is the recalibrated spectra obtained after lock‐mass calibration.
B Create an automatic processing method and custom script:
  1. In *DataAnalysis*, open any *.d* file (any liquid chromatography–tandem mass spectrometry [LC‐MS/MS] data acquired)
  2. Under *Calibrate* → *Parameters* → *Mass List*, choose *Sum Peak*.
  3. Under *Calibrate* → *Parameters* → *Calibration* → *Lock Mass Calibration* → *Calibration group*, select *ESI*.
  4. Click *Edit Lists*
  5. *Compass Reference Mass List Editor* window will open. To create a new reference list, include the name of the reference compound, the ion formula, charge state (*z*), and the exact mass of the ion (Fig. A4‐1).
  6. Under *File* → *Save As*, make sure the file extension is *.ref* (e.g., *Reserpine.ref*) and click *Save*.
  7. *Calibrate* → *Parameters* → *Calibration* → *Lock Mass Calibration* → *Calibration group* and choose the new reference list and set the *Intensity threshold* to 500.
  8. Click *OK*.
  9. Under *Method* → *Script…*, write the following simple *Visual Basic* script: option explicit Analysis.ApplyLockMassCalibration true Analysis.Export “C:\Users\username\userfolder”, daMzXML, daLine Form.close This script will (1) apply lock‐mass calibration using the reference signal selected in the method parameters (e.g., reserpine), (2) export line spectra in *.mzXML* format to the folder specified (e.g., “C:\Users\username\userfolder\”), and (3) close the processed file in DataAnalysis so another file can be processed. Note: No changes will be saved to the raw data file.
  10. Under *Method* → *Save as…*, save the modified method in a recognizable folder.
  11. Under *Tools* → *ProcessWithMethod*, two windows will open. *Compass Compass Automation Engine* and *Compass DataAnalysis ProcessQueuer*.
  12. In *Compass Automation Engine*, click on *Method* to choose the method that was previously saved in the known folder. Then click on *Select* to choose the *raw .d* files that are going to be processed.
  13. Once you have chosen the desired files for processing, click on *Process* to begin. Chosen files should move to *Compass DataAnalysis ProcessQueuer*. During the processing period, DataAnalysis will be busy and a great deal of the computer processing power will be occupied. We recommend running the conversion process overnight and avoiding other computationally intensive processes while data conversion is taking place. In our case, it was usually left for overnight processing.
  14. When the process is complete, the exported *.mzXML* files will be found in the assigned folder.

## Appendix 5 Global Natural Products Social (GNPS) Molecular Networking bioinformatics workflows using MS‐Cluster.

The following protocol uses mass spectra in the construction of a molecular network using the GNPS Molecular Networking online platform (http://gnps.ucsd.edu/ProteoSAFe/static/gnps-splash.jsp; Wang et al., 2016). For a general GNPS manual, see https://bix-lab.ucsd.edu/display/Public/Molecular+Networking+Documentation (Wang et al., 2016). The protocol provided here can be used to repeat networking parameters that we have used to generate networks of foliar metabolites for forest tree communities and other plant samples. After calibration and data conversion to the *.mzXML* file format (see Appendix 4), mass spectra files are organized using “group mapping” and “attribute mapping” files in *.txt* format. Mass spectra files, a group mapping file, and an attribute mapping file are uploaded onto the platform using an ftp client. Once uploaded, user‐generated mass spectra files are selected for networking, and public spectral libraries are chosen against which to compare user‐generated spectra. Finally, networking and library‐matching parameters are chosen for the construction of a molecular network.

A Software required
  1. An ftp client such as WinSCP 5.95.5 or FileZilla 3.27.1
  2. An account on the GNPS Molecular Networking online platform (http://gnps.ucsd.edu)
B Upload mass spectra data to GNPS using an ftp client
  - Setting a new connection to GNPS using *WinSCP*
    1. In *WinSCP* under *login* → *New Site*
    2. Under *Session* → *File protocol*, choose *FTP* → Encryption, choose *No encryption*
    3. Under *Host Name*, write *ccms‐ftp01.ucsd.edu* → *Port Number*, choose *21*.
    4. Navigate to the *GNPS* platform (http://gnps.ucsd.edu) and register as a new user.
    5. Return to *WinSCP*. Under *login* → *Session* → *User Name*, write your GNPS username. Under → *Password*, write your password.
    6. Click *Save*, write site name → Click *Ok*
  - Data upload using *WinSCP*
    1. In *WinSCP* under *login,* choose the directory that you created.
    2. Click *Login*
    3. *WinSCP* will show two window schemes: the local directory (*C:\Users\XXXX*) at left and GNPS server (*/<root>*) at right.
    4. Choose the folder of your files in the local site menu. Highlight the files or folders to upload and select *Upload* by right clicking or drag and drop the file to the GNPS server window on the right. You will then see the files queued and transferred to GNPS.
C Molecular network assembly
  1. Sign in on *GNPS* (http://gnps.ucsd.edu). Then navigate to → *Data Analysis*. Click the highlighted text ‘*Data Analysis*’ to navigate to a new window, the *Network Workflow*, shown in Fig. A5‐1.
  2. Under *Workflow Selection* → *Title*, write the name of the network. We recommend including one's username, date, and some details regarding the parameters used to create the network.
  3. Do not use *Networking Parameter Presets*.
  4. To input your files, navigate to *Basic Options* → *Select Input Files*. A pop‐up window with three tabs will appear: *Select Input Files, Upload Files, Share Files*, as shown in Fig. A5‐2.
  5. Select the *Select Input Files* tab. Proceed by selecting the files or folder that comprise the input mass spectra (typically .mzXML files) and selecting one of *Spectrum Files* G1 through *Spectrum Files G6*.
    - If the spectra can be meaningfully organized into six groups, for example, if the spectra were derived from six plant species, then the spectra representing each group can be uploaded into the six *Spectrum Files* folders. Proceed to Step 6.
    - If the network will contain more than six groups, add all user‐supplied input mass spectra files to *Spectrum Files G1*.
    - Select the files you want to use for *Group mapping*, followed by selecting the *Group mapping* buttons, and then select the files to use for *Attribute mapping*, followed by selecting the *Attribute mapping* buttons.
    - A .txt *Group mapping* file must be provided by the user. This file must be custom‐edited using a text editor (e.g., Notepad++ for Windows or TextWrangler/BBEdit for Mac).
    - To create a *Group Mapping* file use the following format: GROUP_GroupName1=file1.mzXML;file2.mzXML GROUP_ GroupName2=file3.mzXML;file4.mzXML Where “GroupName1” can be any user‐defined group name (e.g., PsychotriaAcuminata, or psycac, or BCI), and “file1.mzXML,” etc. are user‐generated tandem mass spectrometry (MS/MS) spectra files. Each line in the *Group mapping* file must begin with the prefix “GROUP_” in all capital letters.
    - Note: The downstream GNPS network analyses provided in Appendix 6, below, do not depend on groups defined in the *Group mapping* file, but rather assume that filenames include a six‐character species code with which to identify spectra.
  6. Select *Finish Selection* to return to the *Network Workflow* page.
  7. To adjust parameters that govern the sensitivity of MS‐Cluster (under‐the‐hood software that generates consensus MS/MS spectra), molecular networking and spectral library searches, navigate to *Basic Options*.
    - Under *Basic Options* → *Precursor ion mass tolerance*, set the parameter to a value from 0.0075 to 2.0 Da; the lower value represents lower ppm error tolerance. The particular setting of this parameter depends on the mass accuracy of the mass spectrometer as well as the specific instrument method used to collect the MS/MS data. We recommend using a precursor ion mass tolerance value below 1 Da for data collected on a quadrupole‐time‐of‐flight (q‐TOF) instrument.
    - Under *Basic Options* → *Fragment Ion Mass Tolerance*, set the parameter to a value from 0.0075 to 2.0 Da. This value specifies within what range fragment ion *m/z* values will be considered equivalent. We recommend using values below 0.5 Da for data collected on a q‐TOF instrument.
  8. To adjust parameters governing the molecular network similarity matrix alignment and the formation of links between nodes, navigate to *Advanced Network Options* (see Fig. A5‐3).
    - To adjust the threshold of similarity that must occur between a pair of consensus MS/MS spectra, set the value for *Minimum cosine score* to a value between 0.5 and 0.99. The default value is 0.7. Lower values will increase the size of the clusters due the clustering of less similar MS/MS spectra, and higher values will generate smaller clusters and leave more nodes unlinked. We recommend a value ≥0.6 for *Minimum cosine score*.
    - To adjust the maximum number of links to other nodes permitted for any single node, set the value for *Network TopK*. The default value is 10. The edge between two nodes is kept only if both nodes are within each other's *TopK* most similar nodes. We use the default value.
    - To adjust the minimum number of MS/MS spectra permitted to form a consensus spectrum, set the value for *Minimum Cluster Size* to a value ≥1. Make sure that *Run MsCluster* is activated (set to *yes*). MSCluster merges nearly identical MS/MS spectra into consensus spectra that represent structurally unique molecules (Frank et al., 2008). We use values from 1 to 2 depending on the number of replicates collected per sample.
    - To modify the number of common fragment ions compared between two spectra, set the value for *Minimum Matched Fragment Ions*. The default value is 6. We use values from 3 to 6 depending on the molecular weight of molecules.
    - To adjust the maximum size of nodes allowed in a single connected network, set the value for *Maximum Connected Component Size (Beta)*; the default value is 100. We use the default parameter for small networks of one to 10 samples. For large networks (more than 10 samples), we allow an unlimited number of nodes in a single network by setting *Maximum Connected Component Size* to 0.
  9. To adjust the parameters that govern dereplication, or the matching of the user's spectra with those of known compounds in public spectral libraries, navigate to *Advanced Library Search Options*.
    - To control the number of shared fragment ions required for a library match, set the value for *Library Search Min Matched Peaks* to a value ≥6. To control the minimum cosine similarity score required for a library match, set the *Score Threshold* to a value ≥0.6. If the annotation of molecular families is desired, set the *Enable analog search* setting to *Do Search* and choose a value for the upper threshold for the mass difference tolerated between user and library spectra under *Maximum Analog Search Mass Difference*. We use a threshold of 100 Da for plant extracts.
  10. We do not use other advanced parameters for plant extracts. To set other advanced parameters, refer to Molecular Networking Documentation (https://bix-lab.ucsd.edu/display/Public/Molecular+Networking+Documentation).
    1. Click *Submit*. The workflow will begin to process the network in a new window showing a diagram that highlights each task as it is processed. A notification will be emailed to the user‐defined email when the network is complete. Fig. A5‐4 illustrates the *Job Status* window during the execution of a GNPS networking run.
    2. Note that during a run or after the completion of a run, the *Clone* button can be used to return to the *Network Workflow* page (Fig. A5‐1) with all parameters set to the values used for the cloned run.
    3. Upon completion of the network, the *Job Status* window will appear as in Fig. A5‐5.
    4. To download the network data, navigate to the *Status* section and the *Auxiliary Views* header and select *View Network, Node Centric*. A new page will open.
    5. Under *Download tab*, choose *Download*. Then select *Tab‐Delimited Results Only* and *All fields*.
    6. Click the *Download* button. This will download your data as a compressed folder titled “ProteoSAFe‐METABOLOMICS‐SNETS‐[code].zip.”

## Appendix 6 Calculation of chemical structural‐compositional similarity (CSCS) for all pairwise combinations of samples using Global Natural Products Social (GNPS) Molecular Networking output.

A Data organization during liquid chromatography–mass spectrometry (LC‐MS) and GNPS analyses
  1. Downstream analyses using the GNPS network will be facilitated by the consistent application of filenames to MS files during the collection of LC‐MS data. We use a five‐digit numeric code to uniquely identify each sample, followed by a six‐character code to refer to all plant species in the data set, followed by a three‐character code for treatment combinations in analyses concerning intraspecific variation. Other information retained within filenames can be appended after these codes, but should be as consistent as possible. For example, a file containing the results of an LC‐MS run on a sample of *Psychotria acuminata* that was a young, expanding leaf collected during the wet season in the shade would be named “00001_psycac_ywh_20170430.mzXML.”
  2. Blanks should contain the work “blank” in the filename in place of the species code. For example, “00002_blank_20170430.mzXML.”
  3. Load all of the MS sample files to be networked into a single folder on GNPS. This can be a subdirectory of the user directory. For example, “username/forestchem/00001_psycac_ywh_20170430.mzXML.”
B Preparation of an MS‐sample index file using regular expressions
  1. GNPS output will be stored in a zip file titled “ProteoSAFe‐METABOLOMICS‐SNETS‐[code]‐view_network.zip,” where [code] is an eight‐character code generated by GNPS to give the network a unique ID. Move the file to the intended work directory and unzip it.
  2. Place within the working directory a .csv file named “FreshMass.csv” containing three columns: (1) “Sample,” five‐digit unique sample codes, such as 00001; (2) “Species,” six‐character species code; and (3) “FreshMass,” sample masses recorded during Step 2 of the chemical extraction described in Appendix 3.
  3. Use a text editor with the ability to execute search‐and‐replace using regular expressions such as TextWrangler or BBEdit (https://www.barebones.com/products/textwrangler/). We recommend Haddock and Dunn (2011; http://practicalcomputing.org/) for a tutorial on the use of regular expressions to modify text files. Here, we refer to TextWrangler.
  4. Open the file ProteoSAFe‐METABOLOMICS‐SNETS‐[code]‐view_network/params.xml in TextWrangler.
  5. Navigate to Search > Find
  6. Select “Case sensitive” and “Grep.” Ensure that “Selected text only” is not selected.
  7. In the “Find” field, enter: (<parameter name=“upload_file_mapping”>)(spec‐\d\d\d\d\d.mzXML)(.username/forestchem/)(0\d\d\d\d_blank.+mzXML)(</parameter>\r)
  8. Leave the “Replace” field blank.
  9. Select “Replace All.”
  10. In the “Find” field, enter: (<parameter name=“upload_file_mapping”>)(spec‐\d\d\d\d\d.mzXML)(.username/forestchem/)(0\d\d\d\d)(_)(\w\w\w\w\w\w)(.+mzXML)(</parameter>)
  11. In the “Replace” field, enter: \2\t\4\5\6\7\t\6\t\4
  12. Select “Replace All.”
  13. Delete all rows that did not convert (Lines 1–27, ending with the lines “<parameter name=“tolerance.PM_tolerance”>2.0</parameter>” and the final four to 20 lines, including those that identify public MS libraries, for example “<parameter name=“upload_file_mapping”>lib‐00000.mgf|speclibs/MASSBANK/MASSBANK.mgf</parameter>”
  14. In Line 1, add the column headers, separated by tabs: SpecCode, OrigFilename, Species, Sample
  15. Save the file as “SampleSpecMap.txt” in the directory ProteoSAFe‐METABOLOMICS‐SNETS‐[code]‐view_network
C Assembly of the sample chemical composition matrix
  1. Run the function “molecNetsTraits” (Appendix S1), using as input:
    - code, the 8‐character code for the GNPS network
    - date, the date in the format “yyyymmdd”
    - outfile, an output filename, default “MolecNetsChemTraits[date].RData”
  2. The script will write the file “AttributesBlanksRemoved[date].txt,” which includes all attribute data, excluding all network nodes (consensus spectra) observed in blanks. This is useful for generating network figures, for example, using Cytoscape.
  3. The script will write the files “NetworkBlanksRemoved[date].txt,” and “NetworkBlanksRemovedNoSingletons[date].txt.” These files contain the edges of the network and can be used to generate network figures, for example, using Cytoscape. These files will also be used in Step D.
  4. The function “molecNetsTraits” will generate the following objects and save them to the file “MolecNetsChemTraits[date].RData”:
    - sampsByCompounds: rows are leaf samples, columns are network nodes/consensus spectra representing unique compounds, entries are ion intensity
    - sppByCompounds: rows are species, columns are network nodes/consensus spectra representing unique compounds, entries are mean ion intensities of each compound in each species
    - network: the network output given by GNPS; each row is a link between two compounds, columns include CLUSTERID1, CLUSTERID2, and Cosine.
D Calculation of CSCS similarity metric
  1. Run the function “calcCSCS” (Appendix S2), using as input:
    - the date in the format “yyyymmdd”
    - species (TRUE/FALSE); if FALSE, the function calculates CSCS for all pairs of samples, if TRUE, the function calculates CSCS for all pairs of species
    - outfile
    - either sampsByCompounds or sppByCompounds to calculate CSCS for pairs of samps or species, respectively
    - network
  2. The function will generate the following objects and save them to the file “CSCS[date].RData”:
    - sampsCompsStand, standardized relative ion intensity of compounds in each sample or species
    - diag, a diagonal matrix containing each species CSCS similarity to itself
    - cscs, a CSCS chemical similarity matrix for all pairs of samples or species
